# Supplementary material for: RAB5A expression is a predictive biomarker for trastuzumab emtansine in breast cancer
Source: Nat Commun. 2021 Nov 5;12:6427. doi: 10.1038/s41467-021-26018-z (PMC8571323; doi:10.1038/s41467-021-26018-z)
Supplement: Supplementary file 1 — Supplementary Information [file 41467_2021_26018_MOESM1_ESM.pdf]

Supplementary information

RAB5A expression is a predictive biomarker for trastuzumab emtansine in breast cancer

Olav Engebraaten[1,2], Christina Yau[3], Kristian Berg[2, 4], Elin Borgen[5], Øystein Garred[5], Maria E.B. Berstad[2], Ane S.V. Fremstedal[2,], Angela DeMichele[6], Laura van't Veer[3], Laura Esserman[3] and Anette Weyergang[2*]
[1]Department of Oncology, Oslo University Hospital, Oslo, Norway and Institute of Clinical Medicine, University of Oslo, Norway.
[2]Department of Radiation Biology, Institute for Cancer Research, Norwegian Radium Hospital, Oslo University Hospital, Norway
[3]Department of Surgery, University of California San Francisco, California, United States
[4]Section of Pharmaceutics and Social Pharmacy, Department of Pharmacy, University of Oslo, Norway.
[5]Department of Pathology, Oslo University Hospital, Oslo Norway
[6]Department of Medicine, Perelman school of Medicine, University of Pennsylvania, United States

*Correspondent author
Address: Oslo University Hospital, Norwegian Radium Hospital, Ullernchausseen 70, 0379 Oslo, Norway

Content: Supplementary Table 1

Supplementary table 1

Qualifying biomarker analysis results including the lower (LCI) and upper (UCI) 95% confidence intervals for the coefficients (Coef) graphically depicted in 4B.

Statistics Model 1C and 1D describes the Biomarker x Treatment Interaction without (C) and with (D) adjusting for HR status. LR: Likehood ratio

pCR ~ Biomarker																
	TDM1+Pertuzumab (n=52)				Paclitaxel + Trastuzumab (n=31)				Biomarker x Treatment Interaction (n=83)							
	Model 1A				Model 1B				Model 1C				Model 1D			
	Coef	LCI	UCI	LRp	Coef	LCI	UCI	LRp	Int_Coef	LCI	UCI	Int_LRp	Int_Coef_HRAdj	LCI	UCI	Int_LRp_HRAdj
RAB11A	0.06	-0.47	0.59	0.83	0.06	-0.74	0.87	0.88	0.00	-0.96	0.95	0.99	0.00	-0.97	0.98	1.00
RAB4A	0.43	-0.18	1.12	0.17	-0.03	-0.86	0.86	0.94	0.46	-0.61	1.52	0.39	0.45	-0.65	1.53	0.41
RAB5A	0.71	0.15	1.40	0.01	-0.57	-1.67	0.38	0.24	1.29	0.17	2.54	0.02	1.28	0.10	2.60	0.03

	THP (n=44)			
	pCR ~ Biomarker			
	Coef	LCI	UCI	LRp
RAB11A	**-0.79**	**-1.72**	**-0.02**	**0.04**
RAB4A	0.00	-0.57	0.57	1.00
RAB5A	0.38	-0.36	1.17	0.32
