# Supplementary material for: RAB5A expression is a predictive biomarker for trastuzumab emtansine in breast cancer
Source: Nat Commun. 2021 Nov 5;12:6427. doi: 10.1038/s41467-021-26018-z (PMC8571323; doi:10.1038/s41467-021-26018-z)
Supplement: Supplementary file 3 — Reporting Summary [file 41467_2021_26018_MOESM3_ESM.pdf]

nature research

Corresponding author(s): Anette Weyergang

Last updated by author(s): Aug 9, 2021

Reporting Summary

Nature Research wishes to improve the reproducibility of the work that we publish. This form provides structure for consistency and transparency in reporting. For further information on Nature Research policies, see our Editorial Policies and the Editorial Policy Checklist.

Statistics

For all statistical analyses, confirm that the following items are present in the figure legend, table legend, main text, or Methods section.

n/a	Confirmed	
☐	☒	The exact sample size (*n*) for each experimental group/condition, given as a discrete number and unit of measurement
☐	☒	A statement on whether measurements were taken from distinct samples or whether the same sample was measured repeatedly
☐	☒	The statistical test(s) used AND whether they are one- or two-sided<br>*Only common tests should be described solely by name; describe more complex techniques in the Methods section.*
☐	☒	A description of all covariates tested
☐	☒	A description of any assumptions or corrections, such as tests of normality and adjustment for multiple comparisons
☐	☒	A full description of the statistical parameters including central tendency (e.g. means) or other basic estimates (e.g. regression coefficient) AND variation (e.g. standard deviation) or associated estimates of uncertainty (e.g. confidence intervals)
☐	☒	For null hypothesis testing, the test statistic (e.g. *F*, *t*, *r*) with confidence intervals, effect sizes, degrees of freedom and *P* value noted<br>*Give P values as exact values whenever suitable.*
☐	☒	For Bayesian analysis, information on the choice of priors and Markov chain Monte Carlo settings
☒	☐	For hierarchical and complex designs, identification of the appropriate level for tests and full reporting of outcomes
☒	☐	Estimates of effect sizes (e.g. Cohen's *d*, Pearson's *r*), indicating how they were calculated

Our web collection on statistics for biologists contains articles on many of the points above.

Software and code

Policy information about availability of computer code

Data collection	No software was used for data collection
Data analysis	No new codes were generated for this study. The I-SPY 2 biomarker analysis is performed using R v3.6.3. R is publically available, and commands and packages in R were used to do the analysis. In terms of specific packages that are needed, the likelihood ratio test requires the lmtest package (v.0.9-37); the combination of p values to identify the optimal threshold needs the metap package (v1.4); and the Bayesian analysis needs the rjags package (v.4-10). The dataanalysis for the in vitro experiments as well as the Kamilla study was done with the SigmaPlot extended graph analysis 14 software (Systat). ImageLab 4.1 (Bio-Rad) software was used for detection and quantification of western blots. The Gen5 software version 2.09 (Biotek) was used for processing the spectrophotometer data in the cytotoxicity experiments. Image J version 1.53C was used for processing and inserting the scale bar of the IHC images.

For manuscripts utilizing custom algorithms or software that are central to the research but not yet described in published literature, software must be made available to editors and reviewers. We strongly encourage code deposition in a community repository (e.g. GitHub). See the Nature Research guidelines for submitting code & software for further information.

Data

Policy information about availability of data

All manuscripts must include a data availability statement. This statement should provide the following information, where applicable:

- Accession codes, unique identifiers, or web links for publicly available datasets
- A list of figures that have associated raw data
- A description of any restrictions on data availability

All raw data necessary to interpret, verify and extend the research in this article is provided in the data source files.

Field-specific reporting

Please select the one below that is the best fit for your research. If you are not sure, read the appropriate sections before making your selection.

- [x] Life sciences
- [ ] Behavioural & social sciences
- [ ] Ecological, evolutionary & environmental sciences

For a reference copy of the document with all sections, see nature.com/documents/nr-reporting-summary-flat.pdf

Life sciences study design

All studies must disclose on these points even when the disclosure is negative.

Sample size

No sample size calculation was performed for this study. The sample sizes are not based on a statistical method, but based on accessible tumor samples. Thus, no selection of samples from the available patient material has been performed. The sample size for the I-SPY2 samples was dictated by the number of patients enrolled when the arms graduated (Multicenter Adaptive trial, se more information bellow in this paragraph). The sample size for the Kamilla was dictated by the number of patients enrolled at the Radium Hospital when the study was finalized (multicenter study). This is biomarker study includes subsets of patients from two clinical studies. The design considerations of the full clinical trials are not within our control.

For the I-SPY2 study, a total of 127 patients where included; 52 in the TDM1+P group, 31 in the Trastuzumab group and 44 in the trastuzumab +P group. While accrual is ongoing, a statistical engine assesses the accumulating pathological and MRI responses at weeks 3 and 12 and continuously re-estimates the probabilities of an experimental arm being superior to the control in each HR/HER2/MP defined biomarker signature. The efficacy engine uses data from all control arm patients enrolled since the beginning of the trial. Experimental regimens have various sample sizes; and may exit the trial due to futility (predictive probably of phase 3 success < 10% for all signature), graduation (predictive probability of phase 3 success >= 85%), reaching maximal accrual (predictive probability of phase 3 success between 10% and 85%), or as recommended by the I-SPY 2 DSMB (for safety). For the Kamilla study, 24 patients were included in the trials at Oslo University Hospital. Biopsy material from primary breast cancer surgery were available from 19 patients, and all were included in the study.

The statistics has been performed on all available samples from both clinical trials. The two studies described in the article were not performed with the intension to analyze Rab5A as a predictive marker for the clinical outcomes. On this basis, the sample selection was completely unbiased in both studies and only based on the criteria described in the clinical study protocol.

Data exclusions

No data exclusions were done with the I-SPY2 data. For the Kamilla data, 5 patients enrolled in the study at the Norwegian Radium Hospital where not included as no primary biopsy tissue were available for the study

Replication

All in vitro experiments were replicated twice (n=3). For the I-SPY2 and Kamilla data, replication is not applicable. These are clinical trials where each patient can only be assigned to receive therapy on one experimental arm after which they either respond to treatment or not. Replicates of the microarray data /IHC data from each patient would probably be possible for some of the patients. However, patient tissue samples are limited and precious, and we consider replication here as ethically problematic.

Randomization

For the I-SPY2 study, biomarker assessments at screening are used to assess eligibility and classify patients into one of eight subtypes based on hormone receptor (HR), HER2-receptor and Mammaprint (high vs. ultra-high) status. The adaptive randomization engine preferentially assigns patients to agents based on continually updated Bayesian probabilities of pCR rates within predefined biomarker signatures; 20% of patients are randomized to the control arm.

Blinding

I SPY 2 is not a double-blind trial. Physicians and patients are aware of the allocated arm. Each agent has very specific safety and management considerations, so for safety and feasibility, the arm allocation is not blinded to the physician. However, no investigator is aware of how many patients are accrued to each arm, nor are they privy to any of the aggregate results until every patient on study has completed therapy and gone to surgery. Over the course of the trial, 16-20 sites were open to enrollment and up to 5 arms were being enrolled simultaneously. Investigators do not have any information about the status of the ongoing arms in the trial. Only the stats team and DSMB have access to the data and see the results of the adaptive randomization.

Kamilla study the investigators were blinded to the group allocations. In detail, the personnel at the Department of Pathology responsible for the staining and scoring of IHC samples, did not have access to the clinical data. The data analysis and correlations was done by researchers not involved in evaluating the IHC.

Reporting for specific materials, systems and methods

We require information from authors about some types of materials, experimental systems and methods used in many studies. Here, indicate whether each material, system or method listed is relevant to your study. If you are not sure if a list item applies to your research, read the appropriate section before selecting a response.

Materials & experimental systems

n/a	Involved in the study	
☐	☒	Antibodies
☐	☒	Eukaryotic cell lines
☒	☐	Palaeontology and archaeology
☒	☐	Animals and other organisms
☐	☒	Human research participants
☐	☒	Clinical data
☒	☐	Dual use research of concern

Methods

n/a	Involved in the study	
☒	☐	ChIP-seq
☒	☐	Flow cytometry
☒	☐	MRI-based neuroimaging

Antibodies

Antibodies used	For western blotting: HER2 (#2165) antibody from Cell Signaling Technology, RAB5A (PA5-29022), RAB11A (71-5300) and RAB4A (PA3-912) antibody from Thermo Fisher Scientific, γ-tubulin (#T6557) from Sigma-Aldrich, HRP linked anti-rabbit (#7074) and anti-mouse (#7076) were from Cell Signaling Technology. For IHC: RAB5A from Abcam (ab 109534; rabbit IgG, clone EPR5438.
Validation	All antibodies has been validated for the human species as described on the manufactories web sites for the specific antibodies. The HER2-, Rab5A- (Thermo Fisher Scientific), Rab4A-, Rab11A-, γ-tubulin- and HRP linked anti-rabbit- and anti-mouse- antibodies has been verified for the Western blotting procedure as described on the manufactories web sites for the specific antibodies. The Rab5A antibody from Abcam has been verified for IHC as described on the manufactory web side.

Eukaryotic cell lines

Policy information about cell lines

Cell line source(s)	AU-565 (CRL-2351), HCC1954 (CRL-2338), MDA-MB-453 (HTB-131) and SKOV-3 (HTB-77) and MDA-MB-231 (HTB-26) where all provided by ATCC. SK-BR-3 originally obtained from ATCC, were provided by the Department of Biochemistry at Institute for Cancer Research
Authentication	The cell lines from ATCC were all used between passage number 3 and 25 to avoid changes in the cell lines characteristics with time. No authentication were done for these cell lines. Authentication of SK-BR-3 has been done by the Genotype core facility at Oslo University hospital. The sample submitted was processed as follows: DNA from the 10^6 to 10^7 cells submitted was extracted using the Qiagen MagAttract DNA Mini M48 Kit by an automated procedure on the BioRobot M48. The Powerplex 16 kit (Promega, Nerliens Meszansky) was used to amplify the thirteen core CODIS short tandem repeats (STR) loci plus Penta E, Penta D and the gender-determing locus, amelogenin. Alleles are determined by capillary electrophoresis on a Megabace 1000 followed by data analysis with the software Genemarker HID software. The appropriate positive and negative controls were used throughout the analysis and data provided by ATCC was used as a template. After this, the cells has been used between passage number 2 and 25.
Mycoplasma contamination	All cells lines were routinely checked for mycoplasma infections with negative result.
Commonly misidentified lines (See ICLAC register)	No commonly misidentified cell lines were used in this study

Human research participants

Policy information about studies involving human research participants

Population characteristics	The population from the S-SPY study included patients 18 years or more, with HER 2 positive invasive breast cancer histologically confirmed at diagnosis measuring at least 2.5 cm by clinical examination or 2.0 cm by imaging, with no evidence of distant metastatic disease. The I-SPY2 population had not been exposed to previous chemo- or radiation therapy. A full description of the population characteristics is submitted with NCOMMS-21-11823, currently undergoing a second evaluation after minor revision (positive review).<br>The population in the Kamilla study included women, 18 years old or more, with HER2-positive recurrent, metastatic or unresectable locally advanced breast cancer. Patients had received a prior anti-HER2 agent and chemotherapy (prior taxane was not required) and had progressed on or after the most recent treatment or within 6 months of completing adjuvant therapy.
Recruitment	Recruitment to both the I-SPY 2 and Kamilla study was done at the involved study sites in connection to the ongoing diagnosis and treatment of each patient. The patient recruitment was fully independent from the present study, and took place several years before this research project was initiated. Thus, there are no biases from recruitment with impact on the results present here.
Ethics oversight	The I-SPY2 study was approved by IRB boards at the participating study sites:<br>*Institution: University of California San Diego Moores Cancer Center<br>Name and address IRB: University of California, San Diego Human Research Protections Program Institutional Review Boards (Attn: Human Research Protections Program (HRPP) Altman Clinical and Translational Institute, Level 2 9452 Medical Center Drive La Jolla, CA 92093)<br>*Institution: Georgetown University Lombardi Cancer Center<br>Name and address: MedStar Health Research Institute-Georgetown University Oncology Institutional Review Board (Medical-Dental Building, SW104, 3900 Reservoir Road NW, Washington, DC 20057)<br>*Institution: Loyola University Chicago Stritch School of Medicine, Cardinal Bernardin Cancer Center<br>Name and address: Loyola University Chicago Health Sciences Division Institutional Review Board for the Protection of Human Subjects (2160 South First Avenue Maywood, IL 60153)<br>*Institution: University of California, San Francisco, Helen Diller Family of Comprehensive Cancer Center<br>Name and address: UCSF Human Research Protection Program Institutional Review Board (490 Illinois Street, Floor 6, San Francisco, CA 94143)<br>*Institution: University of Texas, Southwestern Medical Center Simmons Comprehensive Cancer Center<br>Name and address: UT Southwestern IRB (5323 Harry Hines Blvd. Dallas, TX 75390)<br>*Institution: H. Lee Moffitt Cancer Center and Research Institute<br>Name and address: Chesapeake IRB (3181 SW Sam Jackson Park Road - L106RI Portland, OR 97239-3098)<br>*Institution: Oregon Health and Science University Knight Cancer Institute<br>Name and address: Oregon Health & Science University Research Integrity Office IRB (3181 SW Sam Jackson Park Road - L106RI, Portland, OR 97239-3098<br>*Institution: Mayo Clinic Breast Cancer Center - Rochester<br>Name and address: Mayo Clinic Institutional Review Boards (201 Building, Room 4-60, 200 First St. SW, Rochester, MN 55905)<br>*Institution: University of Pennsylvania, Abramson Cancer Center<br>Name and address: University of Pennsylvania Office of Regulatory Affairs Institutional Review Board (3624 Market St., Suite 301 S, Philadelphia, PA 19104-6006)<br>*Institution: University of Alabama at Birmingham Comprehensive Cancer Center<br>Name and address: The University of Alabama at Birmingham Office of the Institutional Review Board for Human Use (470 Administration Building, 701 20th Street South, Birmingham, AL 35294-0104)<br>*Institution: University of Minnesota, Masonic Cancer Center<br>Name and address: University of Minnesota Human Research Protection Program (MMC 820<br>420 Delaware St. SE, Minneapolis, MN 55455-0392)<br>*Institution: University of Colorado Cancer Center<br>Name and address: Colorado Multiple Institutional Review Board (COMIRB) (University of Colorado, Anschutz Medical Campus, 13001 E. 17th Place, Building 500, Room N3214, Aurora, CO 80045)<br>*Institution: University of Washington Medical Center<br>Fred Hutchinson Cancer Research Center (FHCRC) IRB (Institutional Review Office<br>1100 Fairview Ave. N. Mail Stop J2-100, Seattle, WA 98109)<br>*Institution: University of Southern California, Norris Comprehensive Cancer Center<br>University of Southern California Health Sciences Institutional Review Board (LAC+USC Medical Center, General Hospital Suite 4700, 1200 North State Street, Los Angeles, CA 90033)<br>*Institution: University of Texas, M.D. Anderson Cancer Center<br>University of Texas MD Anderson Cancer Ctr Clinical IRBs (Office of Human Subjects Protection<br>Unit 1637, 7007 Bertner Ave., Houston, TX 77030-3907)<br>*Institution: Swedish Cancer Institute<br>Western Institutional Review Board (WIRB) (1019 39th Avenue SE Suite 120, Puyallup, WA 98374-2115)<br>*Institution: University of Arizona, Arizona Cancer Center at UMC and UMC-North<br>University of Arizona Institutional Review Board (The University of Chicago Biological Sciences Division/University of Chicago Medical Center, 5751 S. Woodlawn Ave., 2nd floor, Chicago, IL 60637)

The present study using the Kamilla samples was approved by the institutional research review board (Oslo University Hospital, Department of Cancer, Po Box 4953 Nydalen, 0424 Oslo), and the Regional Committees for Medical and Health Research Ethics (REC North-Secretariat, University of Tromo, Po Box 6050 Langnes, 9037 Tromsø).

Note that full information on the approval of the study protocol must also be provided in the manuscript.

Clinical data

Policy information about clinical studies

All manuscripts should comply with the ICMJEguidelines for publication of clinical research and a completedCONSORT checklist must be included with all submissions.

Clinical trial registration

The clinical trial registration number for I-SPY2: NCT01042379. For the Kamilla trial The clinical data was extracted from the 24 patients included at Oslo University Hospital (NCT01702571)

Study protocol

For the I-SPY2 study the redacted protocol that contains the master protocol is submitted as appendix to NCOMMS-21-11823, currently under 2. evaluation by Nature Communications after suggested minor revission (positive review). For the Kamilla study, see primary publication for more information: Eur J Cancer. 2019 Mar;109:92-102. doi: 10.1016/.j.ejca.2018.12.022. Epub 2019 Jan 29. PMID: 30708264.

Data collection

For I-SPY2 Clinical data was collected at each site (see list bellow) using the Electronic Data Capture system, and reviewed by central research coordinators for completeness and accuracy. Data review was under the auspices of QuantumLeap Healthcare Collaborative, the sponsor of I SPY 2. The TH control arm enrolled patients from 3/2010 to 1/2014; and the TDM1/P and THP arms enrolled patients from 6/2013 to 8/2015. The data was collected at the following sites:
University of California San Diego Moores Cancer Center, La Jolla, CA, United States
Georgetown University Lombardi Cancer Center, Washington, DC, United States
Loyola University Chicago Stritch School of Medicine, Cardinal Bernardin Cancer Center Maywood, IL, United States
University of California, San Francisco, Helen Diller Family of Comprehensive Cancer Center, San Francisco, CA, United States
University of Texas, Southwestern Medical Center Simmons Comprehensive Cancer Center, Dallas, TX, United States
H. Lee Moffitt Cancer Center and Research Institute, Tampa, FL, United States
Oregon Health and Science University Knight Cancer Institute, Portland, OR, United States
Mayo Clinic Breast Cancer Center - Rochester, Rochester, MN, United States
University of Pennsylvania, Abramson Cancer Center, Philadelphia, PA, United States
University of Alabama at Birmingham Comprehensive Cancer Center, Birmingham, AL, United States
University of Minnesota, Masonic Cancer Center, Minneapolis, MN, United States
University of Colorado Cancer Center, Aurora, CO, United States
University of Washington Medical Center, East Seattle, WA, United States
University of Southern California, Norris Comprehensive Cancer Center Los Angeles, CA United States
University of Texas, M.D. Anderson Cancer Center, Houston, TX, United States
Swedish Cancer Institute, Seattle, WA, United States
University of Arizona, Arizona Cancer Center at UMC and UMC-North, Tucson, AZ, United States
University of Chicago Medicine Comprehensive Cancer Center, Chicago, IL, United States

For the Kamilla study, the data were collected from the patients included in the study (NCT01702571) at the site Oslo University Hospitaln Norway. The patients were included in the study from April, 2013 to September , 2014. The data were collected at regular study visits, and recorded in the electronic medical records of the patients at the hospital. The data were retrieved directly from the electronic medical records for analysis as described in this study.

Outcomes

The primary endpoint of the patients included from the I-SPY2 trial is pathologic complete response (pCR). pCR was assessed at the time of surgery and is defined as the absence of invasive tumor in breast and reginal nodes (ypT0/is and ypNO). In the event a participant switches to a non-protocol assigned therapy, forgoes surgery, or withdraws from the trial, they are considered "non-pCR" during analysis. The only clinical outcome reported here from the patients from the I-SPY2 study is pCR. Further details on the assessment of outcomes in the I-SPY2 study is submitted with NCOMMS-21-11823, currently undergoing a second evaluation after minor revision (positive review).
For the Kamilla study, the date of first infusion with T-DM1 was recorded as start of the treatment (evaluation) period. The date in the medical record when progression was observed (at clinical or radiological examination) was used to determine the length of progression free survival. Progression free survival was defined as the time period without any progression of disease or death, as determined by regular radiological examinations and clinical follow up. Progression of disease was defined as growth of the evaluated malignant lesions by more than 20% as determined according to RECIST 1.1, or the appearance of new malignant lesions. The only clinical outcome reported here from the patients from the Kamilla study is progression free survival.
